# Integrated PPI- and WGCNA-retrieval of hub gene signatures for soft substrates inhibition of human fibroblasts proliferation and differentiation

**DOI:** 10.18632/aging.204258

**Published:** 2022-09-02

**Authors:** Ziran Xu, Tian Zhou, Yin Wang, Leijie Zhu, Jihao Tu, Zhixiang Xu, Lisha Li, Yulin Li

**Affiliations:** 1 The Key Laboratory of Pathobiology, Ministry of Education, College of Basic Medical Sciences, Jilin University, Changchun 130021, China; 2 The First Norman Bethune Clinical Medical College, Jilin University, Changchun 130021, China; 3 The Third Norman Bethune Clinical Medical College, Jilin University, Changchun 130021, China; 4 Department of Hand and Foot Surgery, The First Hospital of Jilin University, Changchun 130021, China

**Keywords:** soft substrates, fibroblasts, proliferation, PPI, WGCNA

## Abstract

Fibroblasts (FBs) are the most important functional cells in the process of wound repair, and their functions can be activated by different signals at the pathological site. Although wound repair is associated with microenvironmental stiffness, the effect of matrix stiffness on FBs remains elusive. In this study, TGF-β1 was used to mimic the fibrotic environment under pathological conditions. We found that the soft substrates made FBs slender compared with tissue culture plastic, and the main altered biological function was the inhibition of proliferation and differentiation ability. Through PPI and WGCNA analysis, 63 hub genes were found, including GADD45A, CDKN3, HIST2H3PS2, ACTB, etc., which may be the main targets of soft substrates affecting the proliferation and differentiation of FBs. Our findings not only provide a more detailed report on the effect of matrix stiffness on the function of human skin FBs, but also may provide new intervention ideas for improving scars and other diseases caused by excessive cell proliferation, with potential clinical application prospects.

## INTRODUCTION

Pathological scars are pathological products formed during wound healing [1–3]. As a typical fibroproliferative disease [4], its pathological changes are mainly caused by massive proliferation of fibroblasts (FBs) and excessive secretion and deposition of collagen [5]. As the core of scar repair, FBs play an important role in skin wound repair [6], and they may be the key to achieving scar-free healing [7]. After trauma, FBs undergo chemotaxis, proliferate and differentiate into myofibroblasts under the control of cytokines [8]. One of the important reasons for the occurrence of pathological scars is the abnormal over-proliferation of FBs. For example, the imbalance of microenvironmental homeostasis can lead to the abnormal proliferation of FBs, which leads to the formation of keloids [9]. In conclusion, the fate of FBs determines the end state of the scar. Understanding and controlling the biological behavior of FBs is the basis and key to promote wound healing and prevent scarring.

Wound repair is a complex and orderly biological process (BP), which is the result of the interaction and mutual influence of cells, growth factors and extracellular matrix [10]. Under normal conditions, the matrix is involved in maintaining the quiescent state of FBs, and with the increase of matrix stiffness, the proliferation of FBs is gradually activated, which promotes scarring [11]. Earlier studies have found that the stiffness of scar tissue is significantly higher than that of normal tissue, which is the result of scar formation. More and more studies have proved that the changes in the stiffness of the local microenvironment of injury can directly determine the repair and regeneration ability of the wound, and play an important role in the pathogenesis of pathological scars [12–14]. If the wound continues to be stimulated by a large mechanical force, the myofibroblasts continue to proliferate and stimulate the differentiation of myofibroblasts, and the wound will form a pathological scar [15, 16]. At present, most of the treatment methods for pathological scars are based on the principle of inhibiting the inflammatory response. The understanding of the effect of mechanical force signals on scar formation is not deep enough. Therefore, it has become a valuable question whether FBs can be modulated by changing mechanical force or modifying mechanical transduction signals to affect scar formation.

The traditional culture of fibroblast is on tissue culture plastic (TCP) (GPa), the stiffness of its cultural environment is much higher than the physiological environment of FBs [17], this will inevitably affect the properties of FBs, therefore, we constructed the culture conditions with similar stiffness to the normal skin microenvironment (700Pa-1120Pa) [18, 19], explored the mechanical signal-related mechanism of soft substrates affecting the fate of FBs, and tried to provide new ideas and therapeutic targets for the development of pathological scar treatment strategies.

Transforming growth factor β1 (TGF-β1) is a key factor in promoting tissue and organ fibrosis, it regulates the phenotype and function of fibroblasts [20, 21]. And it can also induce the formation of myofibroblasts while inhibit their apoptosis, and promote the deposition of extracellular matrix with collagen as the main component [21, 22]. In severely injured tissue and hypertrophic scar tissue, compared with normal skin tissue, the expression level of TGF-β1 is significantly up-regulated [23, 24], which is considered to be closely related to the formation of pathological scar. Current research suggests that TGF-β1 should be added to *in vitro* experiments so that it can simulate the fibrotic environment under pathological conditions to a certain extent [24], and the results are more meaningful to detect the effect of matrix stiffness on the fate of fibroblasts under this condition.

## RESULTS

### Soft substrates altered the morphology of human foreskin fibroblasts (hFFs)

**Figure 1:**
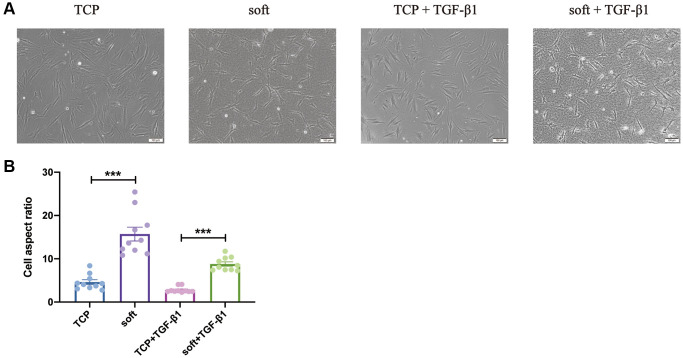
**Soft substrates promoted aspect ratio of hFFs.** (**A**) Phase-contrast images of hFFs on TCP and soft substrates with or without TGF-β1 at day 1. Scale bars: 200 μm (up) or 100 μm (down). (**B**) Aspect ratio of hFFs on TCP and soft substrates with or without TGF-β1 at day 1. ^***^P < 0.001 (mean, *n* = 10).

To explore the effect of soft substrates on FBs, we selected healthy, easily cultured and accessible hFFs, which was developed on either TCP or soft substrates, the elastic modulus of the soft substrates was reported to be 500-1000Pa [25]. At the meantime, the hFFs have been divided into different group based on whether TGF-β1 is added. The cell morphology of the four groups was observed and the aspect ratio was counted, and it was found that after culturing hFFs on TCP and soft substrates with or without TGF-β1, the soft substrates made the boundary of hFFs more distinct, three-dimensional, and slender (Figure 1A), with significant increased aspect ratio (Figure 1B), indicating that the soft substrates had a certain effect on hFFs, and which BPs are specifically affected need further analysis.

### Differential expression analysis

**Figure 2:**
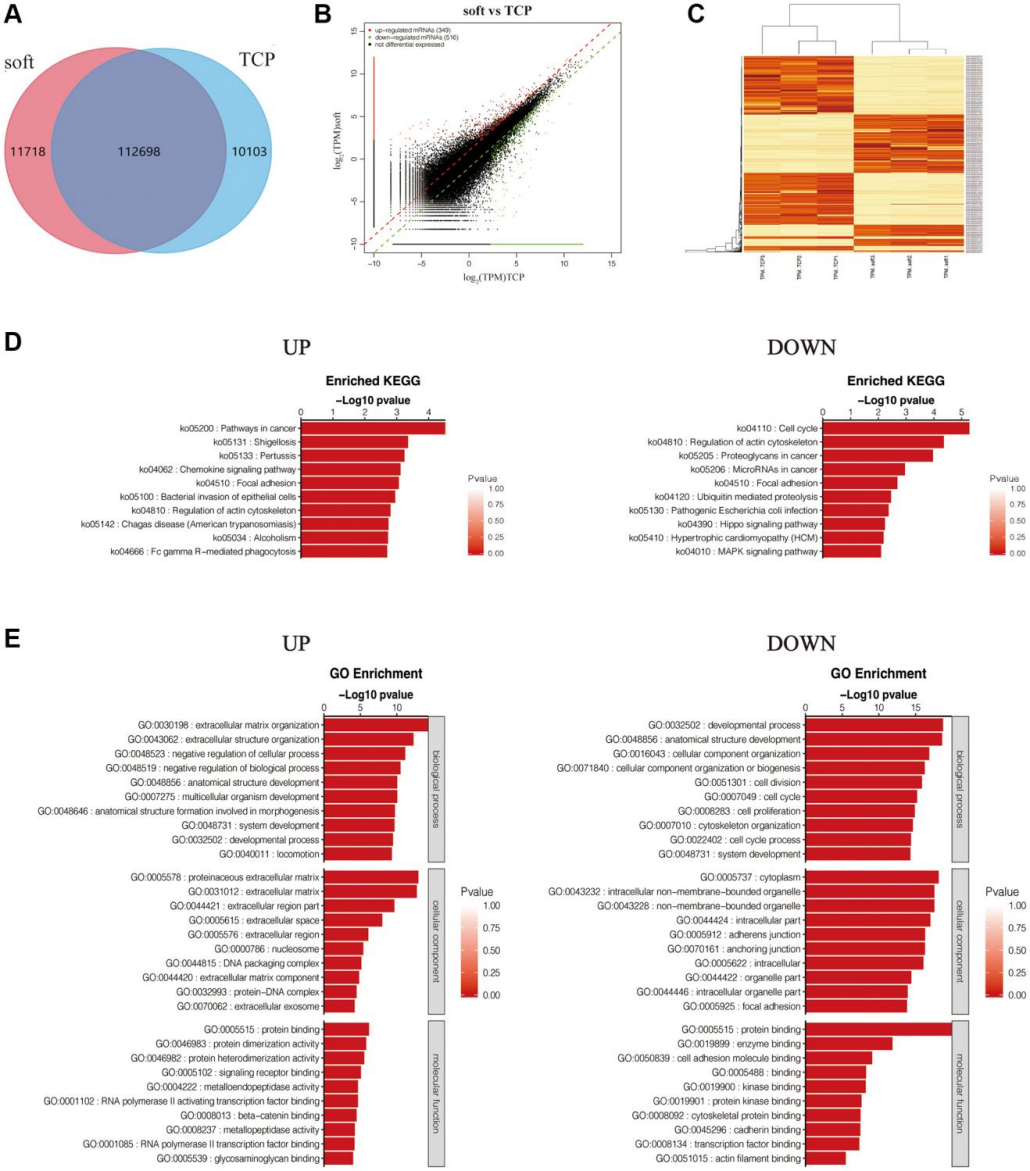
**Differential expression analysis of soft/TCP.** (**A**) Venn diagram of soft and TCP. (**B**) Scatter plot illustrated DEGs of soft/TCP. (**C**) Heat-map cluster analysis of DEGs. (**D**) KEGG pathways of up and down regulated DEGs. The color and horizontal axe were −log_10_ (*p* value) and gene number respectively. (**E**) GO analysis of up- and down- regulated DEGs. The GO analysis categorized mRNA into different groups: BP, CC, and MF. The color and horizontal axe were −log_10_ (*p* value) and gene number respectively.

To explore the BPs and signaling pathways of hFFs that can be altered by soft substrates, we performed mRNA transcriptome sequencing of hFFs cultured on TCP and soft substrates for 24 h, and found that the number of genes co-expressed by soft and TCP was 112,698, the gene expression numbers specific to soft and TCP were 11718 and 10103, respectively (Figure 2A). The number of differentially expressed genes was large, which was not conducive to subsequent analysis. Therefore, the differential expression was screened. It was considered that |log_2_foldchange|≥2 was a significantly differentially expressed gene and was used for subsequent analysis. Among them, the number of up-regulated differentially expressed genes was 349, and the number of down-regulated differentially expressed genes was 516 (Figure 2B). The analysis of the inter-sample repeatability of these genes also found that the repeatability within the group was good, meeting the conditions for subsequent analysis (Figure 2C).

Kyoto Encyclopedia of Genes and Genomes (KEGG) analysis of up-regulated and down-regulated differentially expressed genes (DEGs) showed that the pathways promoted by soft substrates included: pathways in cancer, shigellosis, pertussis, chemokine signaling pathway, focal adhesion, bacterial invasion of epithelial cells, regulation of actin cytoskeleton, chagas disease (American trypanosomiasis), etc. The inhibited pathways included: cell cycle, regulation of actin cytoskeleton, proteoglycans in cancer, microRNAs in cancer, focal adhesion, ubiquitin mediated proteolysis, pathogenic Escherichia coli infection, hippo signaling pathway, etc. (Figure 2D).

Gene Ontology (GO) analysis of up-regulated and down-regulated DEGs showed that the BP promoted by soft substrates included: extracellular matrix organization, extracellular structure organization, negative regulation of cellular process, negative regulation of BP, anatomical structure development, multicellular organism development, etc.; Promoted cellular component (CC) included: proteinaceous extracellular matrix, extracellular matrix, extracellular region part, extracellular space, extracellular region, nucleosome, etc.; Promoted molecular function (MF) included: protein binding, protein dimerization activity, protein heterodimerization activity, signaling receptor binding, metalloendopeptidase activity, RNA polymerase II activating transcription factor binding, etc., BP inhibited by soft substrates included developmental process, anatomical structure development, CC organization, CC organization or biogenesis, cell division, cell cycle, cell proliferation, etc.; inhibited CC included cytoplasm, intracellular non−membrane−bounded organelle, non−membrane− bounded organelle, intracellular part, adherens junction, anchoring junction etc., inhibited MF included protein binding, enzyme binding, cell adhesion molecule binding, kinase binding, protein kinase binding, cytoskeletal protein binding, etc. (Figure 2E). The above results indicated that the effects of soft substrates on hFFs were mainly related to proliferation, differentiation, cytoskeleton and cell-matrix junctions.

### Soft substrates inhibited proliferation and differentiation of hFFs

**Figure 3:**
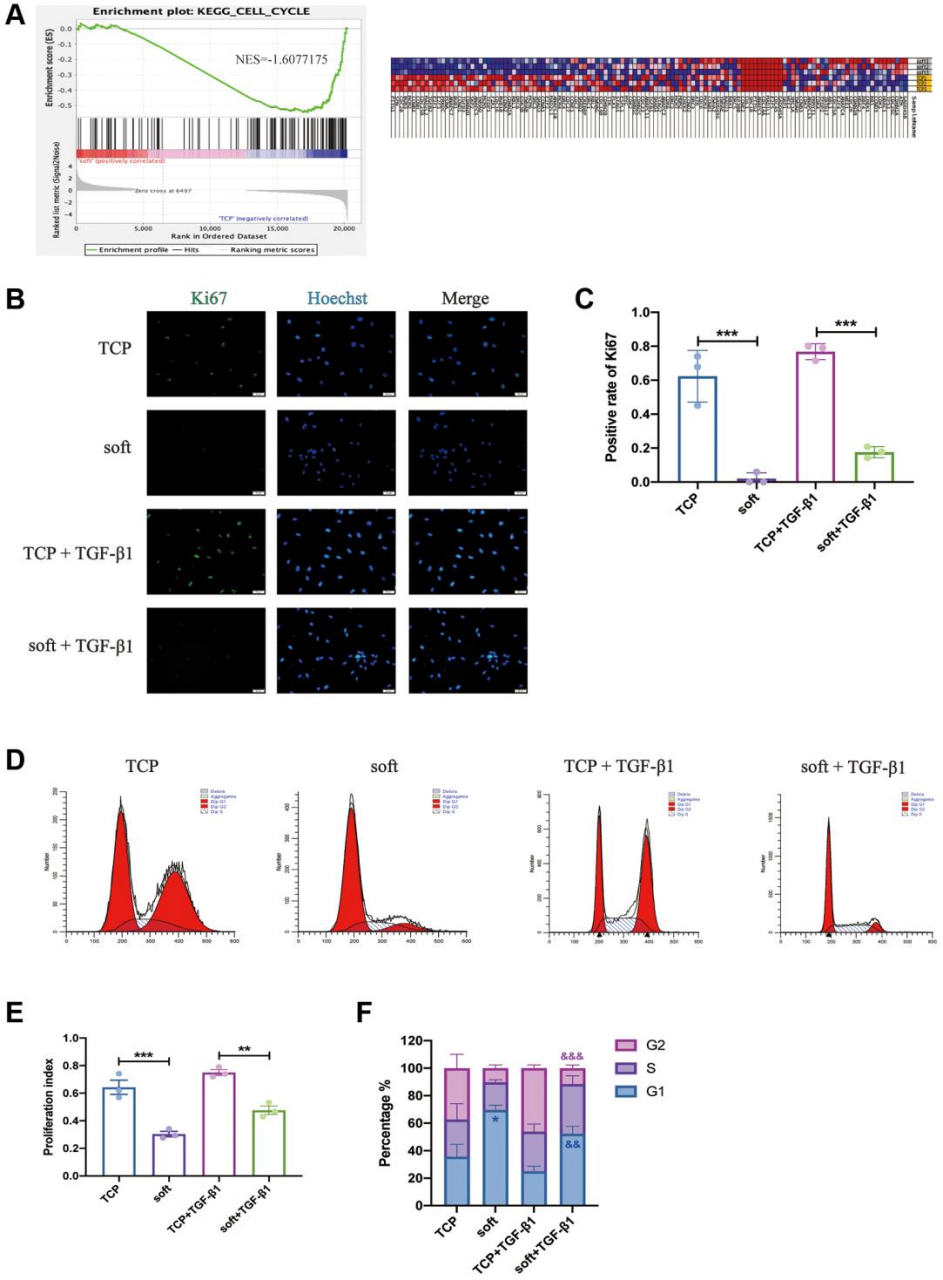
**Soft substrates inhibited the proliferation of hFFs.** (**A**) GSEA for cell cycle of mRNAs in soft/TCP. Enrichment score of cell cycle genes against their expression profile of soft/TCP. The x axis was the level, and the y axis was the enrichment score of these genes (left). Heat map showed the distribution of mRNAs expression of soft/TCP enriched under the cell cycle KEGG entry in all samples under the gene set, with each row representing one sample. Each line represented a gene, the color from blue to red represented mRNA expression from low to high (right). (**B**) Immunostaining against Ki67 of soft and TCP with or without TGF-β1 on day 1. Scale bars: 50 μm. (**C**) The positive rate of Ki67 in B. ^***^P < 0.001 (mean, *n* = 3 randomly selected fields from triplicate samples). (**D**) Detection of cell cycle by flow cytometry for soft and TCP with or without TGF-β1. (**E**) The percentage of PI. ^**^P < 0.01; ^***^P < 0.001 (mean, *n* = 3). (**F**) The percentage of G1-, S-, and G2-phase in the cell cycle. ^*^P < 0.05 vs. TCP; ^&&^*P* < 0.01, ^&&&^*P* < 0.001 vs. TCP+ TGF-β1 (mean, *n* = 3).

In order to explore the main biological effects of soft matrix on hFFs, we carried out gene set enrichment analysis (GSEA) and found that among the GSEA-enriched downregulated entries, cell cycle was the first one, and the enriched genes involved in this entry were shown in the heatmap (Figure 3A). To further validate this result, Ki67 immunofluorescence staining was performed on TCP and soft with or without TGF-β1 (Figure 3B), the results showed that the positive rate of Ki67 in the soft group was much lower than that in the TCP group with or without TGF-β1 (Figure 3C). The cell cycle of the four groups was examined (Figure 3D), and it was found that the soft substrates could reduce the PI of hFFs (Figure 3E), suppress the proportion of cells in G2 phase and increase the proportion of cells in G1 phase (Figure 3F), indicating that soft substrates inhibited hFFs proliferation.

**Figure 4:**
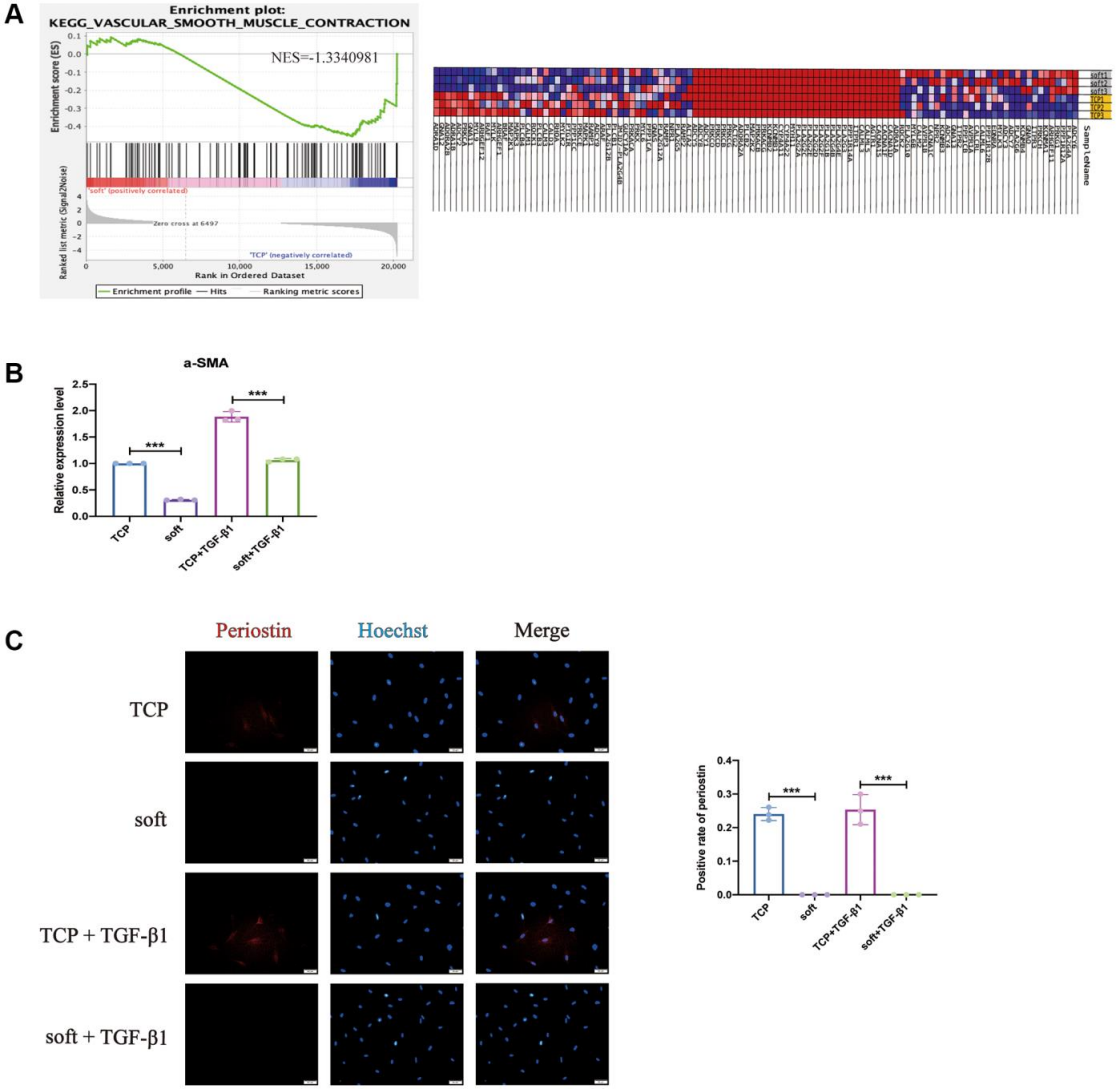
**Soft substrates inhibited contractility of hFFs.** (**A**) GSEA for vascular smooth muscle contraction of mRNAs in soft/TCP. Enrichment score of vascular smooth muscle contraction genes against their expression profile of soft/TCP. The x axis was the level, and the y axis was the enrichment score of these genes (left). Heat map showed the distribution of mRNAs expression of soft/TCP enriched under the vascular smooth muscle contraction KEGG entry in all samples under the gene set, with each row representing one sample. Each line represented a gene, the color from blue to red represented mRNA expression from low to high (right). (**B**) Quantitative reverse transcription PCR (qRT-PCR) analysis comparing *α-SMA* expression levels in soft and TCP. ^***^P < 0.001 (mean, *n* = 3). (**C**) Immunostaining against PERIOSTIN and the positive rate on day 1. Scale bars: 50 μm. ^***^P < 0.001 (mean, *n* = 3 randomly selected fields from triplicate samples).

At the same time, the GSEA analysis also had enriched entries of vascular smooth muscle contraction that interest us, indicating that it had an impact on contractility, including the myofibroblast marker protein α-SMA (also known as ACTA2) [26] (Figure 4A), it was also considered to be one of the most stable cytoskeletal components of myofibroblasts [27], it was found that the soft substrates did significantly down-regulate its expression (Figure 4B). Periostin regulates myofibroblast differentiation and is persistently overexpressed in abnormal scars and other benign fibrous tissues proliferating with fibroblasts [28], while soft substrates Inhibit its expression, also in the presence of TGF-β1 stimulation (Figure 4C), it showed that the soft substrates may inhibit the differentiation and contraction of hFFs, and combined with the soft substrates inhibiting the proliferation of hFFs, these phenomena have certain significance for the treatment of skin scarring, sclerosis, fibrosis and other diseases related to the proliferation of FBs.

### Protein-protein interaction network (PPI) network analysis

**Figure 5:**
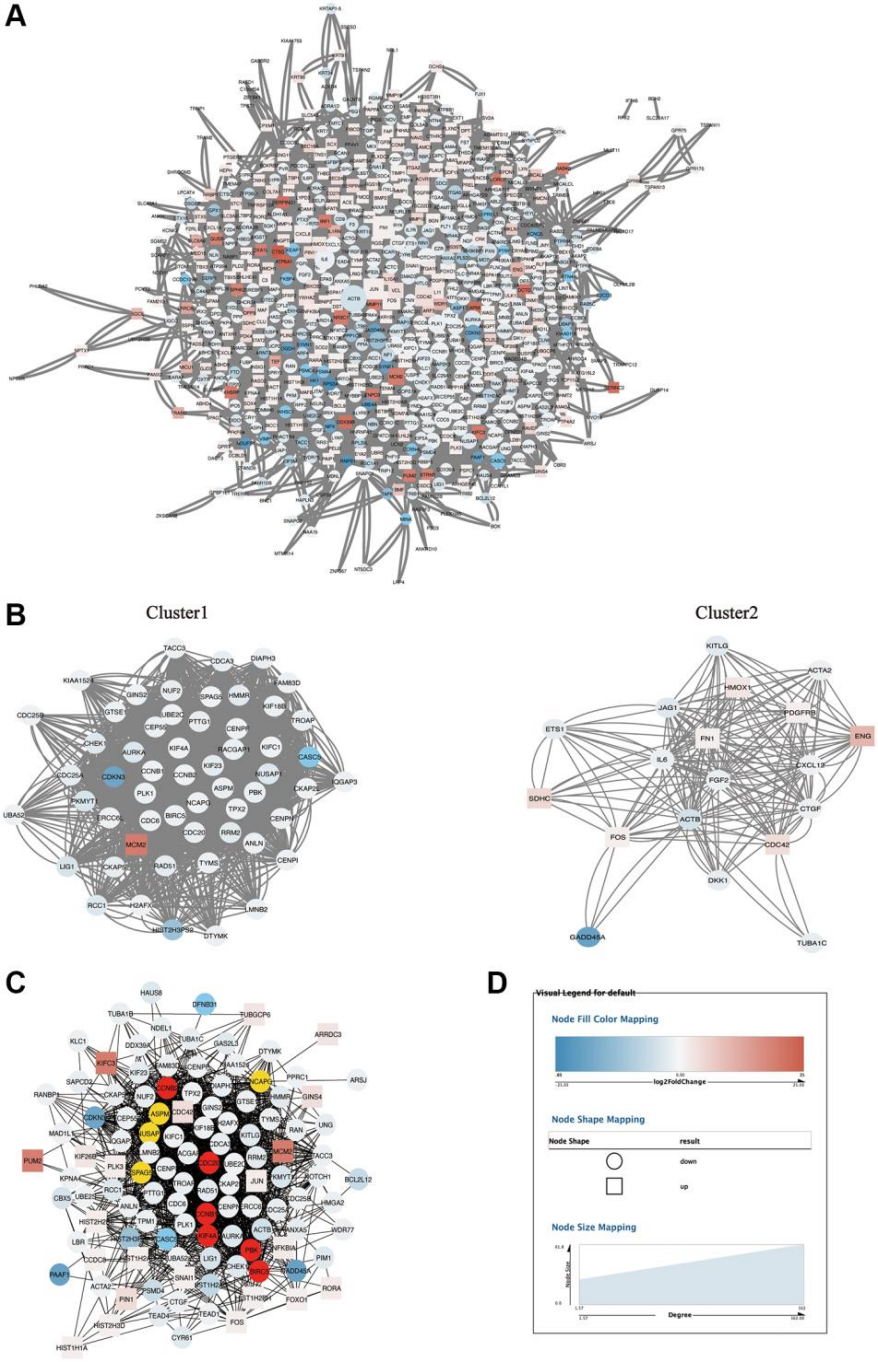
**PPI network analysis of DEGs.** (**A**) The PPI network of 698 DEGs. (**B**) Cluster1 and cluster 2 sub-networks obtained after MCODE analysis of PPI network. (**C**) MCC sub-network obtained after CytoHubba analysis of PPI network. (**D**) The legend of networks. The round represents down-regulated DEGs, the square represents up-regulated DEGs, and the size of the node graph represents the degree for (**A**), which denotes the number of nodes connected to each node. The colors of the nodes indicate the size of log_2_ (fold change). The higher and lower the expression is, the redder and bluer it is, respectively.

In order to explore the PPI network that can significantly change DEGs and select the major sub-networks, we used the string database to construct a PPI network of 698 DEGs and 9602 relations (Figure 5A). Interactions between key genes across the network were determined using Cytoscape plugins (MCODE and CytoHubba). Among them, MCODE obtained the top two clusters, there were 56 nodes and 2160 edges in cluster 1, the enrichment score was 39.273, and there were 19 nodes and 192 edges in cluster 2, the enrichment score was 10.667 (Figure 5B), they were identified from MCODE according to a scoring system. In addition, after importing the PPI network of Figure 5A into another plug-in CytoHubba, 114 key genes were identified by the MCC calculation method, with a total of 1536 edges (Figure 5C). In the above network, the circles represented down-regulated genes, the squares represented up-regulated genes, the blue to red color gradient of node full color corresponds to the log_2_foldchange in the range of -21.03 to 21.03, and the node size (26.02 to 61.80) corresponds to the degree (1.57 to 162) in Figure 5A network (Figure 5D), these genes obtained by MCODE and MCC algorithms were important in DEGs, but the number of genes was large, and further screening was needed.

### WGCNA and meaningful module identification

**Figure 6:**
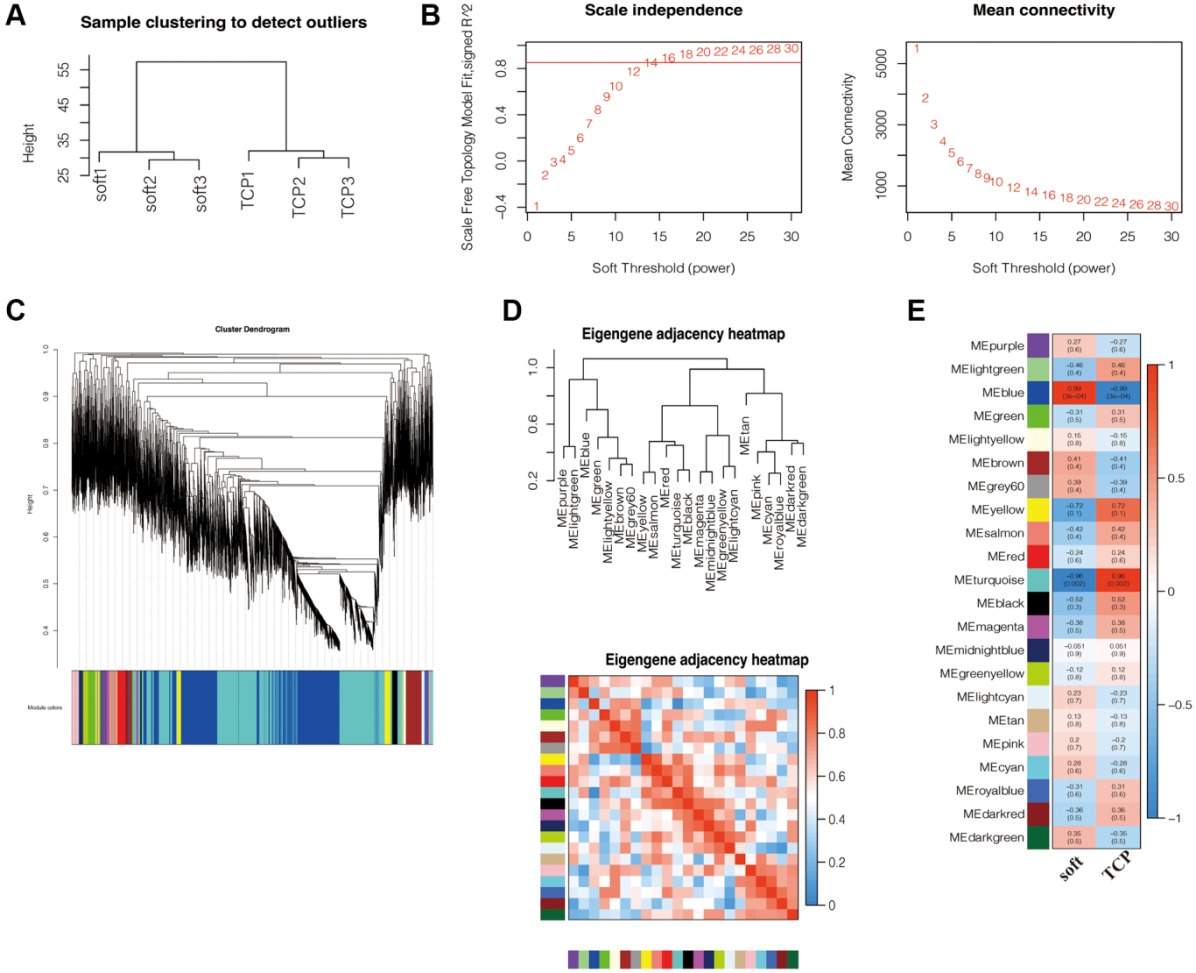
**WGCNA and significant module recognition.** (**A**) Sample clustering found no obvious outliers. (**B**) Analysis of network topology for various soft-thresholding powers. The left panel shows the scale-free fit index (y-axis) as a function of the soft-thresholding power (x-axis). Power 14 was chosen because the fit index curve flattened out upon reaching a high value (> 0.85). The right panel displays the mean connectivity (degree, y-axis) as a function of the soft-thresholding power (x-axis). (**C**) Clustering dendrogram of all mRNAs dataset based on topological overlap. Each module is given a unique colour and represents a cluster of coexpressed genes. (**D**) The eigengene adjacency heatmap was used to illustrate the relationship between eigengenes and phenotypic traits. (**E**) Identification of key modules related to the impact of soft matrices on hFFs. Heatmap displaying the correlations and significant differences between gene modules and samples. Each row corresponds to a module eigengene and each column to a trait. Correlation coefficients and *P* values are displayed in rectangles. Color-coded by relevance based on a color legend. Blue rectangles represent negative correlations between modules and samples, and red rectangles represent positive correlations between modules and samples.

To further screen hub genes, we performed Weighted gene coexpression network analysis (WGCNA) on the transcriptional gene expression profile, sample clustering was performed, and the clustering dendrogram revealed no obvious outliers (Figure 6A), therefore, all samples could be analyzed in the next step. Then, the value of the soft threshold power β was calculated before constructing the gene co-expression network, the scale-free topology threshold of the network was 0.85, and when the soft threshold power was 14, the mean connectivity was close to 0, therefore, β = 14 was chosen to build a hierarchical clustering tree (Figure 6B). Ultimately, 22 modules were identified based on average hierarchical clustering and dynamic tree clipping (Figure 6C). Eigengene adjacency heatmap was used to illustrate the relationship between eigengenes and phenotypic traits (Figure 6D).

The direct relationship between each module and the sample in the transcriptome data, including TCP and soft groups, was analyzed, the results showed that among the modules, the blue module had the strongest correlation with soft (r = 0.99, *P* = 3e-04), including 3215 genes, the turquoise module had the strongest correlation with TCP (r = 0.96, *P* = 0.002), including 3748 genes (Figure 6E), indicating that the genes of blue and turquoise modules might play a role in the effect of soft substrates on hFFs important role, therefore, the blue and turquoise modules were considered as key modules for further analysis.

### Functional analysis and PPI network construction of hub genes

**Figure 7:**
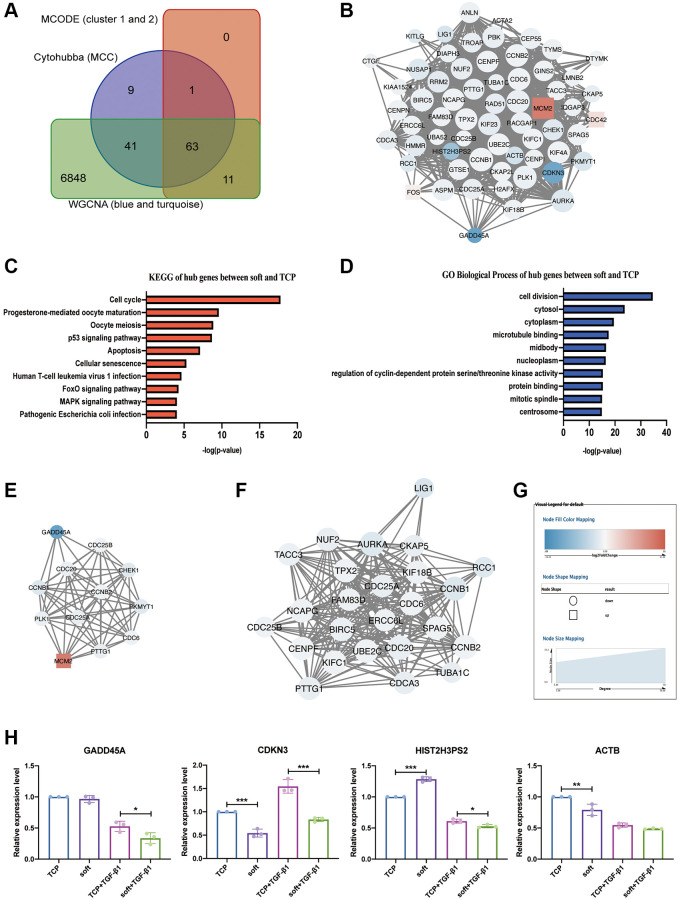
**Screening and analysis of hub genes.** (**A**) Veen diagram of CytoHubba (MCC), MCODE (cluster 1 and 2) and WGCNA (blue and turquoise modules). (**B**) The PPI network of 63 hub DEGs. (**C**) KEGG analysis of 63 hub DEGs. (**D**) GO analysis of 63 hub DEGs. (**E**) The PPI network of 12 hub DEGs in cell cycle. (**F**) The PPI network of 25 hub DEGs in cell division. (**G**) The legend of networks. The round represents down-regulated DEGs, the squar represents up-regulated DEGs, and the size of the node graph represents the degree, which denotes the number of nodes connected to each node. The colors of the nodes indicate the size of log_2_ (fold change). The higher and lower the expression is, the redder and bluer it is, respectively. (**H**) Quantitative reverse transcription PCR (qRT-PCR) analysis comparing *GADD45A, CDKN3, HIST2H3PS2 and ACTB* expression levels in soft and TCP with or without TGF-β1. ^*^P < 0.05; ^**^P < 0.01; ^***^P < 0.001 (mean, *n* = 3).

In order to find more critical hub genes, as well as their relationships and biological functions, we first intersected the 114 genes obtained by the MCC algorithm in CytoHubba, the 75 genes obtained by cluster1 and 2 in MCODE, and the 6963 genes in the blue and turquoise modules of WGCNA, and obtained 63 hub genes (Figure 7A) (Table 1), a PPI network was constructed for them to show their relationship (Figure 7B). KEGG and GO analysis of 63 hub genes found that cell cycle and cell division were the most significant entries (Figure 7C, 7D) (Tables 2, 3), indicating that the most significant effect of soft substrates on hFFs was cell proliferation, and the PPI networks of genes within the entries constructed to represent their relationship (Figure 7E, 7F). In the above network, circles represented down-regulated genes, squares represented up-regulated genes, the blue to red color gradient of node full color corresponded to log_2_foldchange in the range of -21.03 to 21.03, and node size (20.24 to 33.33) corresponded to the value of degree (5 to 58) in the network (Figure 7G), among them, α-SMA (ACTA2) was one of the 63 hub genes, indicating that the main effect of soft substrates on hFFs was to inhibit the proliferation and differentiation of hFFs. At the same time, we further verified the expression of the predicted top-ranked hub genes. Compared with TCP, soft substrates significantly inhibited the expression of *CDKN3* and *ACTB*. In the presence of TGF-β1, soft substrates significantly inhibited the expression of *GADD45A*, *CDKN3* and *HIST2H3PS2* (Figure 7H), which indicated that our predicted hub genes also have certain reliability in the scar hyperplasia model *in vitro*. After a series of analyses, these hub molecules might become therapeutic targets for hypertrophic scars.

**Table 1:** Hub genes for soft substrates effected on hFFs.

**Gene**	**log_2_foldchange**	**Result**	**MCODE_Cluster**	**MCODE_Score**	**Gene name**
GADD45A	−16.30622787	down	Cluster 2	9.61538462	growth arrest and DNA damage inducible alpha
CDKN3	−15.68642757	down	Cluster 1	28.9189189	cyclin dependent kinase inhibitor 3
HIST2H3PS2	−10.13399841	down	Cluster 1	12.675	H3.7 histone
ACTB	−4.069050269	down	Cluster 2	9.86206897	actin beta
LIG1	−3.479187993	down	Cluster 1	17	DNA ligase 1
PKMYT1	−3.339767922	down	Cluster 1	27.2045455	protein kinase, membrane associated tyrosine/threonine 1
NUSAP1	−2.961875051	down	Cluster 1	29.5902547	nucleolar and spindle associated protein 1
AURKA	−2.946966919	down	Cluster 1	29.5902547	aurora kinase A
RCC1	−2.878639248	down	Cluster 1	13.8833333	regulator of chromosome condensation 1
CHEK1	−2.734591706	down	Cluster 1	29.7159091	checkpoint kinase 1
KITLG	−2.620521963	down	Cluster 2	9.23076923	KIT ligand
HMMR	−2.331371536	down	Cluster 1	29.844367	hyaluronan mediated motility receptor
RRM2	−2.326515364	down	Cluster 1	29.5902547	ribonucleotide reductase regulatory subunit M2
KIF23	−2.070150664	down	Cluster 1	29.844367	kinesin family member 23
RAD51	−2.00252251	down	Cluster 1	28.6704545	RAD51 recombinase
CDC25A	−1.892451952	down	Cluster 1	28.5056818	cell division cycle 25A
NUF2	−1.875557123	down	Cluster 1	29.5902547	NUF2 component of NDC80 kinetochore complex
CKAP5	−1.861018053	down	Cluster 1	16.5142857	cytoskeleton associated protein 5
CDCA3	−1.855554867	down	Cluster 1	30.1634921	cell division cycle associated 3
PTTG1	−1.835769591	down	Cluster 1	30.3179487	PTTG1 regulator of sister chromatid separation, securin
TUBA1C	−1.820651131	down	Cluster 2	11.4	tubulin alpha 1c
PBK	−1.746464196	down	Cluster 1	29.5902547	PDZ binding kinase
TROAP	−1.640014332	down	Cluster 1	29.1428571	trophinin associated protein
CDC20	−1.593185081	down	Cluster 1	29.5902547	cell division cycle 20
PLK1	−1.572982533	down	Cluster 1	29.5902547	polo like kinase 1
CEP55	−1.527357106	down	Cluster 1	29.844367	centrosomal protein 55
RACGAP1	−1.460749148	down	Cluster 1	30.0878049	Rac GTPase activating protein 1
ASPM	−1.381827793	down	Cluster 1	29.5902547	assembly factor for spindle microtubules
BIRC5	−1.37934973	down	Cluster 1	29.5902547	baculoviral IAP repeat containing 5
DIAPH3	−1.37772013	down	Cluster 1	22.9233333	diaphanous related formin 3
IQGAP3	−1.358196266	down	Cluster 1	19.7628458	IQ motif containing GTPase activating protein 3
ACTA2	−1.342611936	down	Cluster 2	10	actin alpha 2, smooth muscle
KIF18B	−1.316143376	down	Cluster 1	27.4166667	kinesin family member 18B
ANLN	−1.310215438	down	Cluster 1	28.5064011	anillin actin binding protein
DTYMK	−1.29988096	down	Cluster 1	12	deoxythymidylate kinase
ERCC6L	−1.296164999	down	Cluster 1	27.5483871	ERCC excision repair 6 like, spindle assembly checkpoint helicase
TACC3	−1.295066755	down	Cluster 1	28.0739496	transforming acidic coiled-coil containing protein 3
GTSE1	−1.288618668	down	Cluster 1	28.9585366	G2 and S-phase expressed 1
KIAA1524	−1.276998752	down	Cluster 1	16	CIP2A, cellular inhibitor of PP2A
LMNB2	−1.264456254	down	Cluster 1	14	lamin B2
CENPI	−1.248211164	down	Cluster 1	24.5689655	centromere protein I
TYMS	−1.21959232	down	Cluster 1	28.7837838	thymidylate synthetase
CTGF	−1.217232141	down	Cluster 2	9.40740741	Connective tissue growth factor
FAM83D	−1.165348581	down	Cluster 1	29.4652406	family with sequence similarity 83 member D
CENPN	−1.162540501	down	Cluster 1	30.1142857	centromere protein N
TPX2	−1.140703893	down	Cluster 1	29.5902547	TPX2 microtubule nucleation factor
UBA52	−1.12816062	down	Cluster 4	12	ubiquitin A-52 residue ribosomal protein fusion product 1
NCAPG	−1.116952215	down	Cluster 1	29.5902547	non-SMC condensin I complex subunit G
H2AFX	−1.103617671	down	Cluster 1	18.8190476	H2A histone family, member X
CKAP2L	−1.088642853	down	Cluster 1	29.2941176	cytoskeleton associated protein 2 like
GINS2	−1.088223269	down	Cluster 1	29.6770982	GINS complex subunit 2
SPAG5	−1.081236589	down	Cluster 1	29.5902547	sperm associated antigen 5
CCNB1	−1.071958168	down	Cluster 1	29.5902547	cyclin B1
CCNB2	−1.069246428	down	Cluster 1	29.5902547	cyclin B2
UBE2C	−1.039001281	down	Cluster 1	29.5902547	ubiquitin conjugating enzyme E2 C
KIFC1	−1.033344937	down	Cluster 1	28.7387387	kinesin family member C1
CENPF	−1.032218736	down	Cluster 1	29.5902547	centromere protein F
KIF4A	−1.014116092	down	Cluster 1	29.5902547	kinesin family member 4A
CDC25B	−1.013992092	down	Cluster 1	14	cell division cycle 25B
CDC6	−1.005407699	down	Cluster 1	28.9963415	cell division cycle 6
FOS	1.303245178	up	Cluster 2	9.6	Fos proto-oncogene, AP-1 transcription factor subunit
CDC42	3.298661829	up	Cluster 2	9.97894737	cell division cycle 42
MCM2	16.52548003	up	Cluster 1	28.6770982	minichromosome maintenance complex component 2

**Table 2:** Top 10 terms for KEGG analysis of hub genes involved in soft substrates effected on hFFs.

**Term**	**ID**	**Input**	**Total**	***P*-Value**	**Corrected *P*-Value**	**Enrichment score**
Cell cycle	hsa04110	12	124	1.75E-18	3.64E-16	17.756962
Progesterone-mediated oocyte maturation	hsa04914	7	99	2.58E-10	9.78E-09	9.58838029
Oocyte meiosis	hsa04114	7	128	1.42E-09	4.92E-08	8.84771166
p53 signaling pathway	hsa04115	6	72	2.08E-09	6.67E-08	8.68193667
Apoptosis	hsa04210	6	136	7.70E-08	1.78E-06	7.11350927
Cellular senescence	hsa04218	5	160	5.11E-06	8.24E-05	5.2915791
Human T-cell leukemia virus 1 infection	hsa05166	5	219	2.24E-05	2.79E-04	4.64975198
FoxO signaling pathway	hsa04068	4	132	5.39E-05	5.91E-04	4.26841123
MAPK signaling pathway	hsa04010	5	295	8.95E-05	8.89E-04	4.04817696
Pathogenic Escherichia coli infection	hsa05130	3	55	9.35E-05	9.17E-04	4.02918839

**Table 3:** Top 10 terms for GO analysis of hub genes involved in soft substrates effected on hFFs.

**Term**	**ID**	**Input**	**Total**	***P*-Value**	**Corrected *P*-Value**	**Enrichment score**
Cell division	GO:0051301	25	346	2.36E-35	1.97E-32	34.627088
Cytosol	GO:0005829	42	5095	1.83E-24	7.65E-22	23.73754891
Cytoplasm	GO:0005737	37	4624	2.98E-20	8.30E-18	19.52578374
Microtubule binding	GO:0008017	14	252	3.04E-18	5.07E-16	17.51712642
Midbody	GO:0030496	12	160	3.13E-17	4.35E-15	16.50445566
Nucleoplasm	GO:0005654	31	3630	3.96E-17	4.72E-15	16.40230481
Protein binding	GO:0005515	48	11779	5.28E-16	4.89E-14	15.27736608
Regulation of cyclin-dependent protein serine/threonine kinase activity	GO:0000079	9	55	5.27E-16	4.89E-14	15.27818938
Mitotic spindle	GO:0072686	10	100	1.09E-15	9.06E-14	14.9625735
Centrosome	GO:0005813	15	506	1.31E-15	9.95E-14	14.8827287

## DISCUSSION

After trauma, injury or surgery, FBs in the wound proliferate through mitosis, and their proliferation plays an important role in wound repair in the body. Excessive proliferation of FBs can cause pathological scars and other related diseases, early non-surgical intervention for hypertrophic scarring is the mainstream direction of future development [29].

The increased stiffness of the extracellular matrix is not only the result of fibrosis, but also plays a key role in the processes affecting fibroblast proliferation and matrix synthesis [30]. Studies have shown that the stiffness and other properties of the extracellular matrix have a certain impact on the repair function of FBs, which has potential significance for mesenchymal cell therapy [31]. In fact, many experimental studies have demonstrated that FBs adhere and proliferate more firmly on stiff substrates than soft substrates [30, 32, 33]. In a study of a mouse model of pulmonary fibrosis, the matrix under conditions of normal stiffness is involved in maintaining the quiescent state of FBs [30, 34]. With the increase of matrix stiffness, the proliferation of FBs was gradually activated. In a similar study on mouse cardiac FBs, the experimental results also showed that soft matrix is not conducive to the proliferation and adherence of FBs [35]. In conclusion, matrix stiffness can affect the proliferative activity of FBs [33, 36]. However, the specific mechanism by which extracellular matrix stiffness affects fibroblast proliferation is still unclear.

In this study, it was found through transcriptome analysis that the effect of soft substrates on hFFs mainly inhibited proliferation and differentiation, mainly in that it increased the G1 phase and inhibited the G2 phase of the cell cycle, and inhibited the expression of α-SMA and periostin [28]. In the KEGG pathway analysis, we found that there was enrichment of biological functions in the down-regulated pathways of regulation of actin cytoskeleton and cell cycle, and these pathways were closely related to cell proliferation. In addition, we noticed significant enrichment of actin cytoskeleton and focal adhesion related signaling pathways in both up-regulation and down-regulation. Cytoskeleton is the basic structure of intracellular mechanical force signal transduction [37], and focal adhesion is the structural connecting unit between ECM and actin skeleton [38, 39]. This result suggests that the regulation of ECM stiffness on the fate of FBs relies on the classic pathway of cytoskeleton as a signal transduction pathway. It was particularly noteworthy that we found the existence of Hippo signaling pathway in the downregulation pathway with the top ranking of KEGG analysis results. Hippo pathway has been identified as the key pathway of mechanical stimulation signal conversion and transmission, which is closely related to the proliferation, differentiation and other functions of cell populations [40, 41]. TAP/TAZ is the main effector of Hippo signaling pathway, and it is also the decisive factor for extracellular matrix stiffness to determine the fate and function of fibroblasts [42]. In recent years, studies have proposed that mechanical stimulation signal and Hippo signaling pathway are jointly involved in the regulation of YAP/TAZ activity [43, 44]. Our research results confirm this view to a certain extent. The specific mechanism of this regulation is still unclear, and our results may provide key node genes for revealing the relationship between the two.

In the GO analysis, BP showed that the enriched up-regulated pathways included negative regulation of BP, negative regulation of cellular process, and the down-regulation pathways mainly include cell cycle process, cytoskeleton organization, cell proliferation, cell cycle, and cell division, these results all point to the proliferation function of cells. These results also support that the changes of cytoskeleton may be the reason why soft substrates inhibited FBs proliferation [45, 46].

In order to explore the hub genes affected by soft substrates on hFFs, we analyzed the WGCNA and PPI networks, and found 63 hub genes by taking the intersection. The most important functional enrichment of these hub genes was cell cycle and cell division. There were 12 genes in the cell cycle, among which the log_2_foldchange of *MCM2*, *GADD45A*, *CCNB1*, *CHEK1*, and *PKMYT1* ranked high. The existing literature confirms that the functions of the above genes are all related to cell proliferation [47–51]. There were 25 genes involved in cell division, among which *LIG1*, *CCNB1*, *AURKA*, *RCC1* and *CDC25A* had the highest log_2_foldchange. These genes have also been proved to be involved in the regulation of cell proliferation [49, 52–55]. In addition, 63 hub genes contain α-SMA, which is an important marker of FBs differentiation into myofibroblasts [16, 56]. Considering the critical role of this differentiation process in wound healing and scar formation, matrix stiffness may have an impact on scar outcome by regulating differentiation function.

In summary, our findings demonstrate the effect of soft substrates on the proliferation and differentiation of FBs even in the presence of TGF-β1, and it is expected to apply soft substrates in the treatment of pathological scars, and genes such as *GADD45A*, *CCNB1*, *MCM2*, *CDC25A* have the potential to become new therapeutic targets. Our research also provides new clues for the transformation of hypertrophic scars to atrophic scars, and opens up new ways to explore how to reduce scars or no scars after wound healing.

## MATERIALS AND METHODS

### Cell culture

Normal human FBs were isolated from foreskin tissues of healthy donors (provided by Dr. Lin Chen) [57]. Briefly, these tissues were washed with PBS, cut into 1 mm^3^ pieces. After cells were digested with 0.15% collagenase (Roche Applied Science, USA), the suspension was collected, filtered, centrifuged, resuspended, and then cultured in Dulbecco’s modified Eagle’s medium (Gibco, USA) containing 10% fetal bovine serum (Gibco, USA), 1% NEAA (Gibco, USA), 1% PS, 0.5% GlutaMAX (Gibco, USA) with or without 10 ng/ml TGF-β1 (PeproTech, Inc. USA), at 37°C in a humidified incubator containing 5% CO_2_. The centrifuge tubes were obtained from Jet Biofil Co., Ltd, China. The cells of FBs at passages 3 to 5 were used to further experiment.

### Preparation of the collagen I

Firstly under the condition of ice bath, the amount of solution as described in the following table separately added to the 1.5 mL EP tube and vortexed for 3 min. Add it dropwise in an amount of 300 μl to one well of a 24-well plate (NEST Biotechnology Co., Ltd, China), rotate it slowly for a circle to make it evenly and flatly attached to the bottom of the well plate, incubate at 37°C for 30 min, lay 200 μl of 0.1% gelatin in each well, and incubate at 37°C for 1 h, it could then be used to culture cells.

**Table:** 

**Formula**	**0.1 mol/L PBS**	**4% NaOH**	**H_2_O**	**0.01 mol/L PBS**	**Collagen I (Advanced BioMatrix, 5153)**
soft substrates/ μL	50	2.5	155	84	125

### RNA-seq

RNA of TCP and soft concentration as well as purity was measured using NanoDrop 2000 (Thermo Fisher Scientific, Wilmington, DE, USA). The RNA Nano 6000 Assay Kit of the Agilent Bioanalyzer 2100 system (Agilent Technologies, Santa Clara, CA, USA) was used for assessing RNA integrity.

### Immunofluorescence staining

FBs were cultured in a 24-well plate. First, cells were fixed with 4% paraformaldehyde for 10 min at 4°C. Subsequently, cells were washed with PBS for twice (3–5 min/time) and blocked with 10% BSA for 10 min at room temperature. Then aspirating the blocking solution from the specimen without washing the specimen, FBs were incubated with Ki67 primary antibody (Abcam, Cambridge, UK; Rabbit polyclonal antibody) for 1 h. The FBs were washed with PBS and incubated with anti-rabbit (Invitrogen) away light for 40 min at room temperature. Lastly, Hoechst was used for staining for 10 min at room temperature. Images were obtained on Olympus (FV1000 and FV1200) confocal microscopes.

### Cell cycle detection by flow cytometry

After digestion and centrifugation by trypsin, hFFs are washed with PBS for 3 times, 5 min/time and are resuspended in 75% alcohol precooled with 4°C and fixed at 4°C overnight. The next day, the fixed solution is centrifuged and discarded and hFFs are washed with PBS for 3 times, 5 min/time. After that, the PI/RNase mixture (4087S, CST) is added and incubated for 15~20 min in the dark. Lastly, the reaction solution is discarded after centrifugation and PBS is added into hFFs for detection by flow cytometry. The calculation formula of proliferation index (PI) is: PI = (S + G2/M)/ (G0/G1 + S + G2/M) × 100%.

### RT-qPCR

After washing the cells twice with PBS, pre-cooled Trizol (1 ml/1 million cells) was added, lysed for 5 min, collected in 1.5 ml RNase-free EP tubes, centrifuged at 12,000 rpm for 5 min at 4°C, and the supernatant was added with chloroform of 1/5 Trizol volume, vortex for 1 min, centrifuge at 12000 rpm for 15 min at 4°C, add an equal volume of isopropanol to the supernatant, invert and mix, let stand for 10 min, centrifuge at 12000 rpm for 30 min at 4°C, wash the precipitate with 75% ethanol, centrifuge at 7500 rpm for 5 min at 4°C, and dissolve the pellet with DEPC water after drying. cDNA was obtained according to the instructions of Hifair^®^ II 1^st^ Strand cDNA Synthesis SuperMix for qPCR (gDNA digester plus) kit, and qPCR was performed according to Hieff^®^ qPCR SYBR Green Master Mix (High Rox Plus) kit (α-SMA forward: CCCATCTATGAGGGCTATGC, reverse: CACGCTCAGTCAGGATCTTC).

### GSEA

GSEA was performed based on our mRNA data using the GSEA software version4.2.1. Gene-lists were derived from the mRNA data by ranking them by their expression levels between two samples. Since then, some genes from c2.cp.kegg.v7.5.1.symbols.gmt was mapped to a list of genes arranged in advance to calculate ES score (enrichment), which nPerm 1000 was arranged to calculate significant sex, and determine 2 set of genes that false discovery rate value (FDR) < 0.25 was considered to have significant sex.

### Differential expression analysis

The DESeq2 was used for differential expression analysis between two conditions/groups. Using Benjamini and Hochberg’s approach for adjusting *P* values, the false discovery rate was controlled. Genes with *P*-value < 0.01 were designated as differentially expressed. FDR < 0.01 and fold change ≥2 were the thresholds to determine whether to identify genes with significant differential expression using DESeq2.

### GO enrichment and KEGG pathway enrichment analysis

Based on Wallenius non-central hypergeometric distribution, GOseq R packages analyzed DEG GO enrichment analyses [58], which allowed gene length bias to be adjusted.

KEGG [59] can analyze high-level functions and utilities of cells through molecular datasets (http://www.genome.jp/kegg/). KOBAS [60] software was used to count the enrichment of DEGs in the KEGG pathways.

### PPI

Import the DEGs into the STRING database: http://string-db.org/) to obtain the PPI relationship between these DEGs. Afterwards, the PPI relationships of these DEGs were imported into Cytoscape for visualization [61].

### WGCNA

A total of 95139 transcripts were detected, the six duplicate samples were all 0 removed, the transcripts with the same gene name were merged (average), and then MAD was used for screening. The genes with the top 10,000 median deviation values were selected for one-step WGCNA network construction. In WGCNA, genes with similar expression patterns are classified into the same module, and the first principal component of the module is called the intrinsic gene of the module. Here we convert the experimental group into a 0–1 matrix, and we could estimate the correlation between the module and the trait by the expression of the module’s eigengenes. The correlation mode was set to Pearson correlation.

### Statistical analysis

For statistical analysis, student’s *t*-test was used to compare the differences between two groups, one-way ANOVA Tukey’s multiple comparisons test to compare four groups, two-way ANOVA Tukey’s multiple comparisons test for G2, S and G1 percentage. Quantitative data are presented as mean ± SD of at least three independent experiments. *P*-values < 0.05 were considered as statistically significant.

### Availability of data and materials

The datasets used and/or analysed during the current study are available from the corresponding author (Lisha Li: 43973966@qq.com; lilisha@jlu.edu.cn on reasonable request. Correspondence and requests for materials should be addressed to Ziran Xu (1240300750@qq.com; xuzr18@mails.jlu.edu.cn) and Lisha Li (43973966@qq.com; lilisha@jlu.edu.cn).
